# Research and Statistical Analysis on Impact Resistance of Steel Fiber Expanded Polystyrene Concrete and Expanded Polystyrene Concrete

**DOI:** 10.3390/ma15124216

**Published:** 2022-06-14

**Authors:** Wenlong Huo, Sherong Zhang

**Affiliations:** 1 State Key Laboratory of Hydraulic Engineering Simulation and Safety, Tianjin University, Tianjin 300350, China; 1019205047@tju.edu.cn; 2 School of Civil Engineering, Tianjin University, Tianjin 300350, China

**Keywords:** steel fiber expanded polystyrene concrete, expanded polystyrene concrete, drop-weight test, impact resistance, statistical analysis, energy dissipation

## Abstract

Steel fiber foamed concrete (SFFC) combines the impact resistance of steel fiber concrete (SFC) and the energy absorption characteristics of foamed concrete (FC), and it has brought attention to the impact field. Using the mechanical properties of SFFC expanded polystyrene concrete, we prepared (EPSC) specimens with 10%, 20%, 30%, 40%, 50% by volume of expanded polystyrene (*V*_eps_), and steel fiber expanded polystyrene concrete (SFEPSC) specimens by adding 1% steel fiber (SF) based on the EPSC in this study. The relationship between compressive strength, the *V*_eps_ and apparent density was revealed. The relationship between the first crack and the ultimate failure impact of SFEPSC specimens was obtained by a drop-weight test. The impact resistance of SFEPSC and EPSC and the variation law of *V*_eps_ were studied by mathematical statistics. The log-normal and the two-parameter Weibull distributions were used to fit the probability distribution of impact resistance of the SFEPSC and EPSC specimens. Finally, both types of specimens’ destruction modes and mechanisms were analyzed. The mechanism of the EPS particles and the SFs dissipating impact load energy was analyzed from the energy point of view.

## 1. Introduction

It is well known that concrete structures encounter both static and dynamic loads (such as seismic, shock, and explosion loads) during their design life [1,2,3]. Concrete structures are more likely to be destroyed under the dynamic load, and the casualties and property losses are also more serious [4,5]. To make concrete structures safe, some scholars have researched improving the dynamic mechanical properties of concrete structures [6,7,8]. It has been shown that metal foams with good impact resistance [9,10] are often used as a protective layer of structures. However, the cost of foam metal is high, and it is not suitable for the construction of buildings as a whole. As a low-cost porous material, FC has good energy dissipation properties [7]. The static and dynamic compressive properties, stiffness and toughness of foamed concrete can be significantly improved by adding glass fibers (GF) and polypropylene fibers (PPF) [11,12,13,14]. Therefore, it is more suitable for the direct construction of concrete structures. However, there can be a weakness if the concrete structure is subjected to a high-temperature detonation shock wave; the weak fire resistance of GF and PPF leads to decreased mechanical properties [15,16,17]. Some researchers think that SF is a good material [18]; they hold that SFFC has excellent physical properties [19], dynamic mechanical properties and fire resistance [20,21]. However, these studies revealed the ultimate bearing capacity of SFFC at different strain rates through an SHPB test but ignored the accumulation of fatigue damage during the cyclic impact process. This is extremely detrimental to the protective effect of the material during service and even affects the operational safety of concrete structures in service. Therefore, it is significant to reveal the statistical characteristics of fatigue damage of protective materials under impact loads.

In addition, the type of foaming agent [22] and pore structure [23] also affect the mechanical properties of concrete structures. The mechanical properties of FC were significantly reduced in the dry-wet cycle environment [24]. EPSC, which makes use of the advantages and makes up for the deficiencies of FC, is suitable for above-ground, underground and dry-wet cycle projects [25,26]. However, studies incorporating SF have rarely been reported. In this study, the EPSC specimens with five EPS volumes (*V*_eps_ = 10%, 20%, 30%, 40% and 50%) were designed and marked as S0E10, S0E20, S0E30, S0E40, S0E50. Based on the EPSC, the SFEPSC specimens were prepared by adding 1% SF by volume, marked as S1E10, S1E20, S1E30, S1E40, and S1E50. A drop-weight test statistically analyzed the impact test results of SFEPSC and EPSC. The fatigue damage characteristics and energy dissipation mechanism of two types of concrete materials were also analyzed.

## 2. Experimental

### 2.1. Materials and Mix Proportions

P.O 42.5 grade ordinary Portland cement (PC), whose compressive and flexural strengths are 24.3 MPa (3-d) and 4.1 Mpa (3-d) by the Chinese standard GB/T 17671–1999 test method [27], was used in this study. Microsilica (Ms) can improve the mechanical properties of materials. Thus, the mix proportion of Ms was replaced by 10% of the cement mass. The chemical composition of cement and microsilica is shown in Table 1.

**Figure 1:**
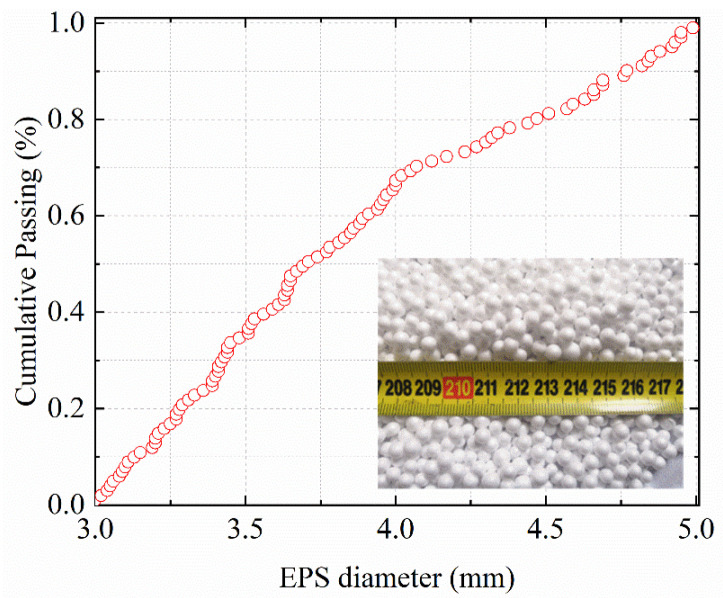
EPS gradation curve.

**Figure 2:**
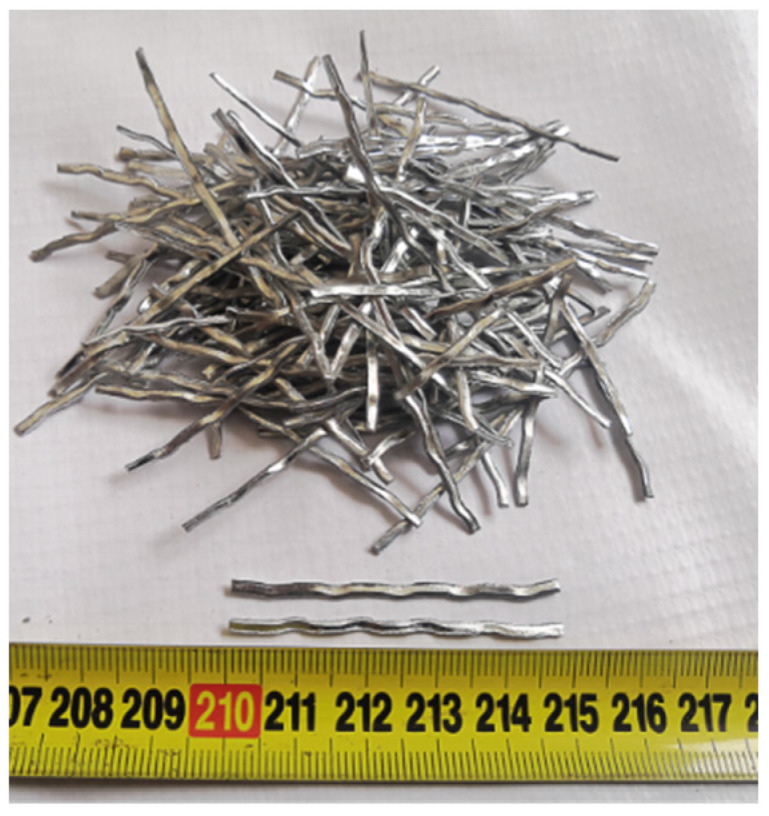
Corrugated steel fiber.

EPS with good heat insulation and shock absorption was prepared by suspension polymerization of styrene and adding a blowing agent. The density of EPS is 25 kg/m^3^, and the diameter is 3–5 mm. Figure 1 shows the diameter gradation of the EPS particles used in the experiment. Corrugated steel fiber (SF) shown in Figure 2 was made of cold-rolled strip steel through a shearing and scoring process, which has high tensile strength, easy dispersion and good adhesion to concrete. The SF has a density of 7810 kg/m^3^, a length of 48 mm, and an aspect ratio (length of SF/diameter or width of SF) of 24. The tensile strength was 610 MPa.

Fine aggregate (FA) was natural river sand with a bulk density of 1710 kg/m^3^ and a medium sand fineness modulus of 2.73. The usage amount was 30% of the aggregate mass. The coarse aggregate (CA) was limestone, and its physical properties are shown in Table 2. Polycarboxylate superplasticizer (PS) was used, for which the water (W) reduction rate was 20–30%.

**Figure 3:**
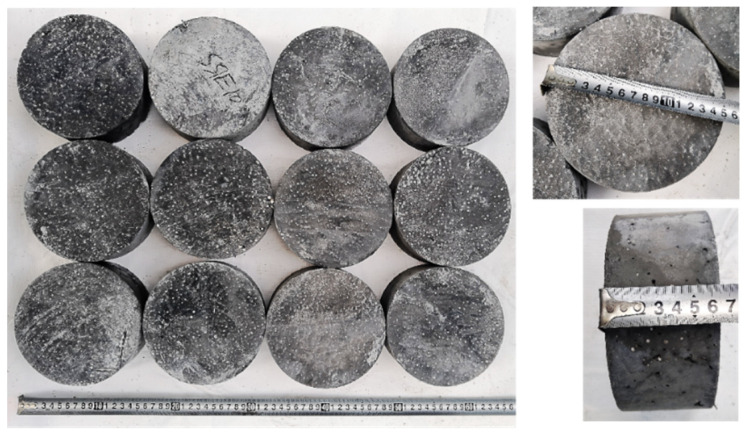
Specimens.

The SFEPSC and EPSC specimens with a diameter of 152 mm and a thickness of 64 mm are shown in Figure 3. There were 12 specimens prepared for each mix proportion, and the total account of both types of specimens was 120. The compressive strength (*f_cu_*) of the basis mix proportion marked S0E0 was 48.7 MPa (28-d). The specimen type number and mix proportion of SFEPSC and EPSC are shown in Table 3. 

### 2.2. The Influence of V_eps_ and Apparent Density on Compressive Strength

**Figure 4:**
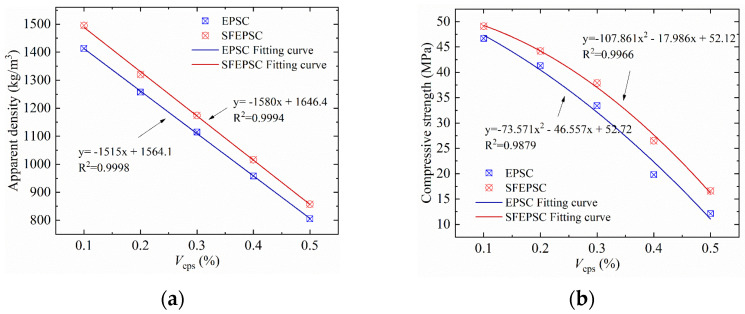

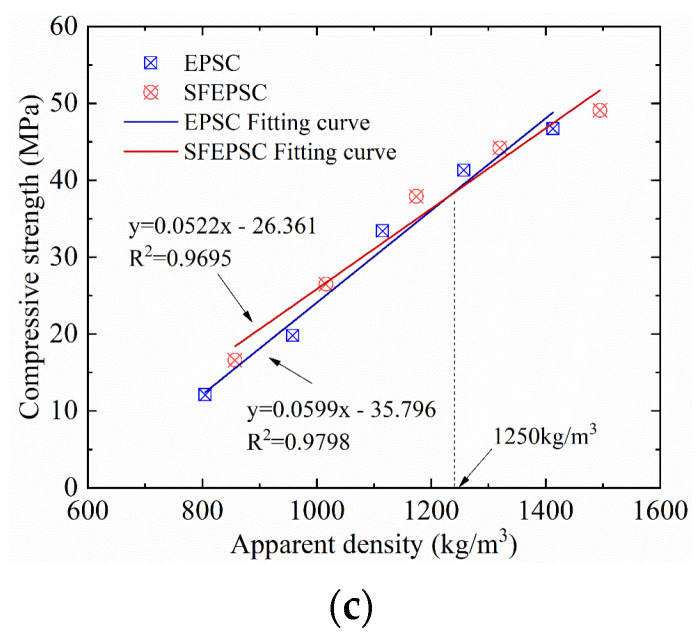
The relationship between compressive strength, the *V*_eps_ and apparent density of SFEPSC and EPSC: (**a**) relationship between apparent density and *V*_eps_; (**b**) relationship between compressive strength and *V*_eps_; (**c**) relationship between compressive strength and apparent density.

Figure 4a,b, and c show the relationship between compressive strength, the *V*_eps_ and the apparent density of SFEPSC and EPSC. It can be seen in Figure 4 that: (1) the apparent density of two types of concrete decreases linearly with the increase in *V*_eps_, as shown in Figure 4a. The apparent density of SFEPSC decreases 4.9% faster than the apparent density of EPSC, and the compressive strength shows a quadratic curve decreasing trend with the increase in *V*_eps_ shown in Figure 4b. (2) The compressive strength of both concrete specimens increases with the apparent density, as shown in Figure 4c. The compressive strength of SFEPSC increases at a slower rate than EPSC when the apparent density is less than 1250 kg/m^3^. When the apparent density is more than 1250 kg/m^3^, the compressive strength increase rate is opposite to that of less than 1250 kg/m^3^. (3) The compressive strength of SFEPSC at *V*_eps_ equal to 10%, 20%, 30%, 40% and 50% are 49.1 MPa, 44.2 Mpa, 37.9 Mpa, 26.5 Mpa and 16.6 Mpa, as shown in Figure 4c. The apparent density is 95%, 84%, 75%, 65% and 55% of S0E0. The compressive strength of EPSC at *V*_eps_ equal to 10%, 20%, 30%, 40% and 50% are 46.7 MPa, 41.3 MPa, 33.4 MPa, 19.8 MPa and 12.1 MPa. The apparent density is 90%, 80%, 71%, 61% and 51% of S0E0. The above results showed that the compressive strength of SFEPSC can be higher than S0E0 when the *V*_eps_ is equal to 10%.

### 2.3. Drop-Weight Test Device and Test Method

**Figure 5:**
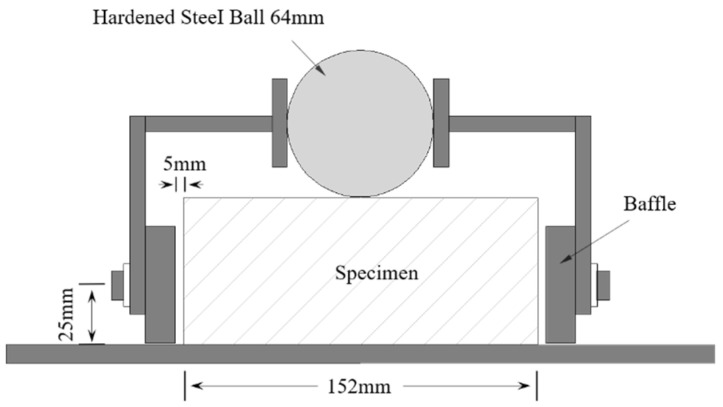
Drop-weight test device.

This experiment adopted the standard test and method recommended by the ACI544 committee [28]. The test device and the specimen placement are shown in Figure 5. We applied lubricating oil to the bottom of the specimens to reduce the friction of the fixed plate during the test process. There were four baffles approximately 5 mm from the edge of the specimen. The steel ball weighed 4.54 kg and was freely dropped from a height of 457 mm. Each impact completed was recorded as a cycle. The surface of the specimen was observed after each impact, and the number (*N*_1_) of first-crack impact resistance in blows was recorded when the first visible crack appeared on the specimen. Impacts continued to occur until the specimen touched three baffles, and the number (*N*_2_) of the ultimate failure impact resistance in blows was recorded, along with the difference number (Δ*N*) of impacts between the first crack (*N*_1_) and the ultimate failure number (*N*_2_).

### 2.4. Test Results and Statistical Analysis

It can be seen that the dispersion of EPSC is higher than SFEPSC from Table 4, which lists *N*_1_, *N*_2_, and Δ*N* of SFEPSC and EPSC in the drop-weight test. About 70% of total EPSC specimens were completely destroyed at the first visible crack, and about 30% could bear the load before the first visible crack. The specimen impact resistance of *V*_eps_ = 50% was about twice that of *V*_eps_ = 10%.

The SFEPSC specimen could continue to bear the impact load after the first visible crack impact. The *N*_1_, *N*_2_ and Δ*N* were higher than EPSC. The SFEPSC specimen, in which the *V*_eps_ was 20%, could still bear the highest load capacity after the first-crack impact, and the average impact resistance was up to 6.7 times greater than S0E20. The above showed that the overall impact resistance of SFEPSC is higher than EPSC.

**Figure 6:**
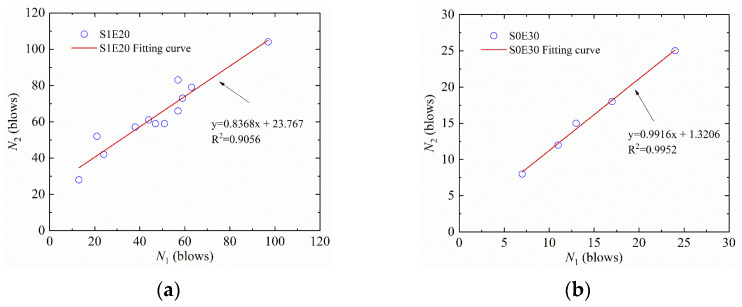
Scatter diagram of impact data with fitted regression line for SFEPSC and EPSC: (**a**) S1E20; (**b**) S0E30.

According to the theory of linear regression analysis, the linear relationship between the first-crack impact resistance in blows and the ultimate failure impact resistance in blows can be regressed by Formula (1):

*N*_2_ = *a* × *N*_1_ + *b* (1)

where *a* and *b* are regression coefficients. The linear regression curves of SFEPSC and EPSC are shown in Figure 6, and the linear regression parameter values are shown in Table 5.

There is a good linear relationship between *N*_1_ and *N*_2_, as shown in Figure 6 and Table 5. If we exclude the data (101/109) of S1E10 in Table 4, then the *R*^2^ =0.8717 becomes *R*^2^ = 0.9105. Therefore, the *R*^2^ =0.8717 can still be used to describe the set of S1E10 specimens. Due to the small amount of EPSC specimen data, the linear relationship could not be well represented. If the data of the EPSC specimens was large enough, their functional relationship could be fully shown. For example, S0E30 and S0E50 both have linear functional relationships.

The mean value ($\bar{x}$), standard deviation (SD) σ, and coefficient of variation ($COV=\sigma /\bar{x}$) of the impact resistance indicators of SFEPSC and EPSC are listed in Table 6.

The fluctuation range of $\bar{x}$, *σ* and COV of SFEPSC corresponding to *N*_1_ and *N*_2_ shows volatility, which is less than 22.8%, as shown in Table 6. It can be seen that both *N*_1_ and *N*_2_ of SFEPSC are inversely proportional to the *V*_eps_ when *V*_eps_ < 30%. The SF and concrete together bear a large amount of load because of the small *V*_eps_, and the specimen showed larger SFC discrete features [29,30]. The fluctuation range of *N*_1_ and *N*_2_ of SFEPSC becomes smaller when *V*_eps_ ≥ 30%, and the overall fluctuation is stable at a constant value. The specimen shows a significant buffering effect at a big *V*_eps_.

Table 6 shows that the overall impact resistance of EPSC is relatively low. When *V*_eps_ is less than 30%, the number of impacts of EPSC is inversely proportional to *V*_eps_. When *V*_eps_ is more than 30%, the fluctuation range of the impact number of EPSC decreases, and the overall value tends to be a constant value. The EPSC has the highest impact resistance at *V*_eps_ = 30%.

*COV* is an important indicator that reflects the degree of data dispersion. A small *COV* value reflects that the data is concentrated near the mean value, and the degree of dispersion is small. On the contrary, a big *COV* value reflects that the data deviates far from the mean value, and the degree of dispersion is large. The *COV* of SFEPSC is smaller than that of EPSC in Table 6, indicating that the impact resistance of SFEPSC is more stable. The ultimate failure specimen proportion at the first-crack impact of EPSC is considerable. Although SD and *COV* are both reduced, the overall impact resistance of the EPSC specimen tends to be stable. This indicates that the impact resistance of both types of concrete specimens decreases with increasing *V*_eps_, and the stability of impact resistance of SFEPSC is better than that of EPSC.

## 3. Probability Distribution Characteristics

**Figure 7:**
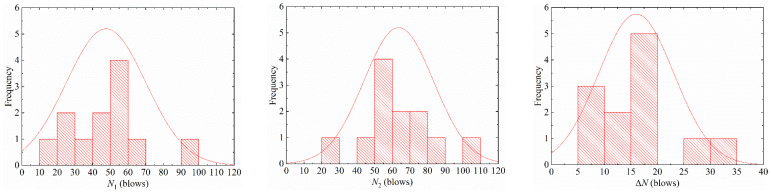
Distribution of the impact resistance for S1E20.

**Figure 8:**
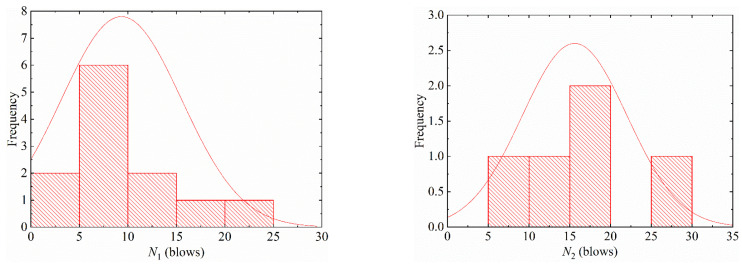
Distribution of the impact resistance for S0E30.

A common method was used to determine the distribution type of specimen statistical data: a certain typical characteristic distribution was used as a hypothesis according to the probability density distribution characteristics of specimen data, followed by hypothesis testing to determine whether it conformed well. This research used statistical analysis methods to perform statistical analysis in Table 4, and the statistical results are shown in Figure 7 and Figure 8. According to the characteristics of the specimen distribution, it is proposed to use log-normal distribution and two-parameter Weibull distribution to fit the probability distribution of impact test results, respectively.

### 3.1. Log-Normal Distribution

**Figure 9:**
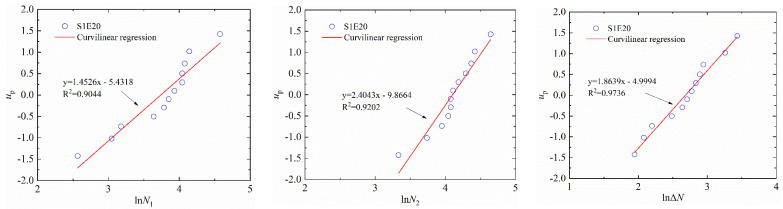
The linear regression of *N*_1_, *N*_2_ and Δ*N* of S1E20 in log-normal distribution.

**Figure 10:**
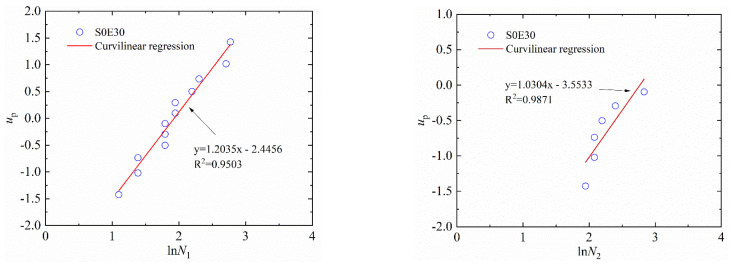
The linear regression of *N*_1_ and *N*_2_ of S0E30 in log-normal distribution.

The normal probability paper test is a commonly used method to test the normality of data [31,32]. The horizontal axis of the normal probability paper is represented by a random variable *X* for a uniform scale. The vertical axis is represented by *F*(*x*) for the non-uniform scale. If the distribution function *F*(*x*) is the normal type, then (*x*, *F*(*x*)) is a straight line on the normal probability paper. The statistic of specimen function plays an important role in statistical inference, and the order statistic is commonly used in reliability research. Suppose that *n* specimens are taken from the population, and they are arranged in ascending order and denoted as *x*_(1)_ ≤ *x*_(2)_ ≤ … ≤ *x*_(*n*)_, where *x*_(*i*)_ is called the *i* order statistic of specimen subset, which is a function of the specimen subset and also a random variable. Called the substandard *i* (*i* = 1, 2, …, n) of *x*_(*i*)_, the rank or order number of $x_{\left(i\right)}$. When the observations are equal, the average value of the substandard *i* is regarded as the rank of these observations. The first-order statistic *x*_(*i*)_ of the specimen subset is the minimum value, and the end order statistic *x*_(*i*)_ of the specimen subset is the maximum value. *F_n_* is written as:

(2) $$F_{n}\left(x\right)=\{\begin{array}{l} 0;\;\;\;\;\;\;x<x_{\left(i\right)}\;\\ \frac{i}{n+i};\;\;\;x_{\left(i\right)}\leq x<x_{\left(i+1\right)}\\ \frac{n}{n+1};\;\;\;x_{\left(n\right)}\leq x \end{array}$$

where *F_n_*(*x*) is the empirical distribution function. According to Bernoulli’s law of large numbers, *F_n_*(*x*) is almost close to *F*(*x*) when *n* is large enough. If (*x*, *F_n_*(*x*)) is drawn in the coordinate system, it should be close to a linear function. The linear relational expression is

(3) $$Y=\alpha_{1}X-\beta_{1}$$

where $Y=u_{p}$, with $u_{p}$ being the cumulative probability density; $X=\ln N_{1}$; and the $\alpha_{1}$ and $\beta_{1}$ are the regression coefficients. For example, the linear regressions of $N_{1}$, $N_{2}$ and Δ*N* of S1E20 and S0E30 in a log-normal distribution are shown in Figure 9 and Figure 10, respectively. Table 7 and Table 8 lists the $\ln N-u_{p}$ linear regression results of SFEPSC and EPSC.

### 3.2. Weibull Distribution

The fatigue life of SFC obeys the Weibull probability distribution [33,34]. The impact resistance of SFEPSC and its fatigue performance are similar in nature to the force mechanism. Therefore, the Weibull distribution analyzed the probability distribution of the impact resistance of SFEPSC in this study. The distribution law of the impact resistance index of two types of concrete specimens can be expressed by the following Weibull density function:

(4) $$f\left(N\right)=\frac{b}{N_{a}-N_{0}}{\left(\frac{N-N_{0}}{N_{a}-N_{0}}\right)}^{b-1}\times \exp\left[-{\left(\frac{N-N_{0}}{N_{a}-N_{0}}\right)}^{b}\right]\;\;\;\;\;\;\;\;N_{0}\leq N<\infty$$

where $N_{0}$ is the minimum life parameter, $N_{a}$ is the characteristic life parameter, and b is the Weibull shape parameter. The Weibull variable is denoted by $N_{\xi }$. According to the Weibull density function f(N) given by Formula (4), the survival rate of the Weibull variable $N_{\xi }$ is obtained. Considering the reliability, the minimum life parameter $N_{0}$ in Formula (4) is taken as 0, which is simplified to the two-parameter Weibull distribution:

(5) $$f\left(N\right)=\frac{b}{N_{a}}{\left[\frac{N}{N_{a}}\right]}^{b-1}\exp\left[-{\left(\frac{N}{N_{a}}\right)}^{b}\right]\;\;\;\;\;0\leq N<\infty$$

Then

(6) $$P\left(N>N_{\xi }\right)=\exp\left[-{\left(\frac{N}{N_{a}}\right)}^{b}\right]$$

**Figure 11:**
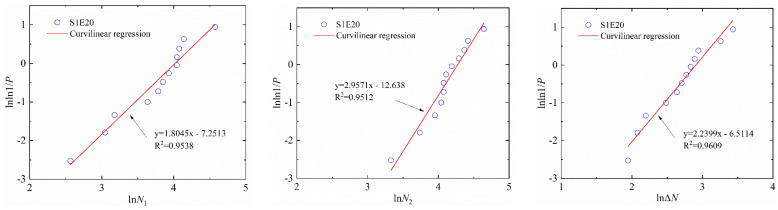
The linear regression of *N*_1_, *N*_2_ and Δ*N* of S1E20 in the Weibull distribution.

**Figure 12:**
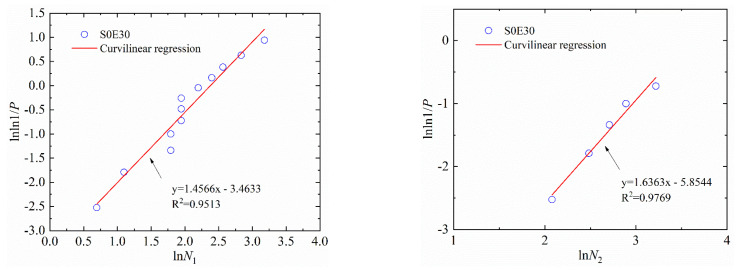
The linear regression of *N*_1_ and *N*_2_ of S0E30 in the Weibull distribution.

Equation (6) is transformed into $\frac{1}{P}=\exp{\left(\frac{N}{N_{a}}\right)}^{b}$, and the logarithm of both sides is obtained:

(7) $$\ln ln\left(1/P\right)=b\ln N-b\ln N_{a}$$

which is

(8) $$Y=\alpha_{2}X-\beta_{2}$$

where Y=lnln(1/P); X=lnN. Here, $\alpha_{2}$ and $\beta_{2}$ are the regression coefficients. Equation (8) can be used to test whether the test data of two types of concrete obey the two-parameter Weibull distribution. For example, the Weibull distributions of the number of impacts of S1E20 and S0E30 are shown in Figure 11 and Figure 12, respectively. Table 9 and Table 10 lists the lnN−lnln(1/P) linear regression results of $N_{1}$, $N_{2}$ and ΔN of SFEPSC and EPSC.

The data points of S1E20 are all near a linear function shown in Figure 9 and Figure 11, which show that both the log-normal distribution and the Weibull distribution can better describe the impact resistance of SFEPSC. The *N*_1_ and *N*_2_ of SFEPSC can be described by the log-normal distribution and the Weibull distribution, as shown in Table 7 and Table 9. Since there were fewer EPSC specimens available for complete failure at the first visible crack (*N*_1_), only the distribution study of the *N*_1_ of EPSC was carried out. The results show that the *N*_1_ of EPSC can be described by two distributions (Figure 10 and Figure 12, Table 8 and Table 10).

### 3.3. Curve of SFEPSC and EPSC Impact Resistance

According to Equations (3) and (8), the corresponding failure probability of the two distributions of SFEPSC and EPSC can be obtained for the number of impact resistance *N*_1_ and *N*_2_.

The log-normal distribution is:

(9) $$N=\exp\left(\frac{u_{p}+\beta_{1}}{\alpha_{1}}\right)$$

The Weibull distribution is:

(10) $$N=\exp\left[\frac{lnln\left(1/P\right)+\beta_{2}}{\alpha_{2}}\right]$$

where $\alpha_{i}$, $\beta_{i}$ are obtained from Table 7, Table 8, Table 9 and Table 10. We calculated the impact resistance performance indexes under different failure probabilities and list them in Table 11 according to Formulas (9) and (10).

**Figure 13:**
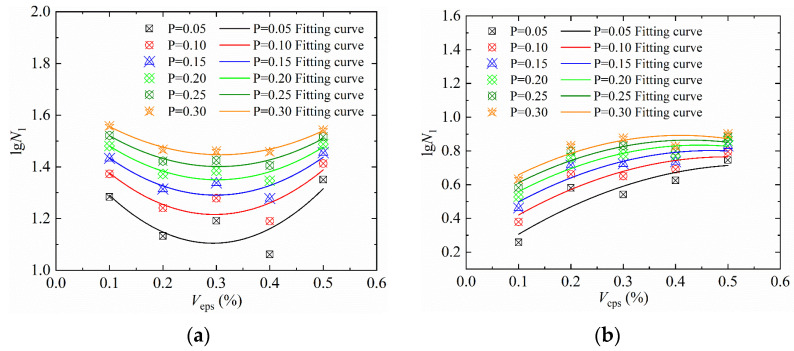
*P* − *V*_eps_ − *lgN*_1_ curves in log-normal distribution: (**a**) SFEPSC; (**b**) EPSC.

**Figure 14:**
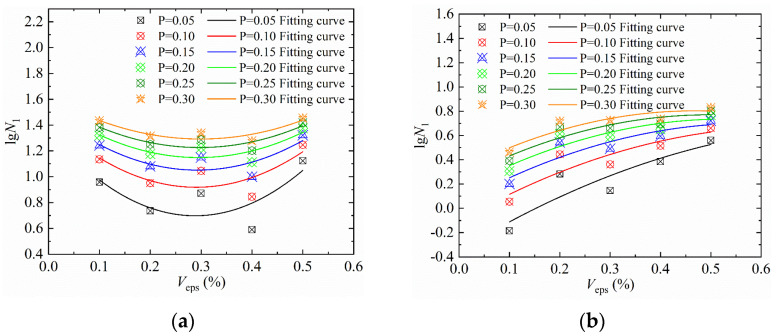
*P* − *V*_eps_− *lgN*_1_ curves in Weibull distribution: (**a**) SFEPSC; (**b**) EPSC.

We then plotted the *P* − *V*_eps_ − *lgN*_1_ curve [31,35] of the impact resistance of SFEPSC and EPSC, as shown in Figure 13 and Figure 14, according to the data in Table 11. The numbers of the first crack of SFEPSC and EPSC and the *V*_eps_ are shown in a conic relationship under different failure probabilities, and the concavity and convexity of the conic relationship are different. The curve normalized fitting is shown in formula (11), and the coefficients *m*, *n* and *l* are shown in Table 12.

(11) $$lgN_{1}=mV_{\mathrm{eps}}^{2}-nV_{\mathrm{eps}}+l$$

## 4. Destruction Mode and Energy Consumption Mechanism

### 4.1. Destruction Mode

**Figure 15:**
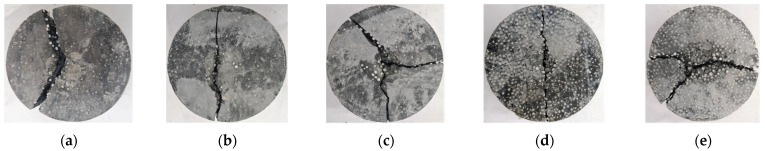
Destruction mode of EPSC specimens: (**a**) S0E10; (**b**) S0E20; (**c**) S0E30; (**d**) S0E40; (**e**) S0E50.

**Figure 16:**
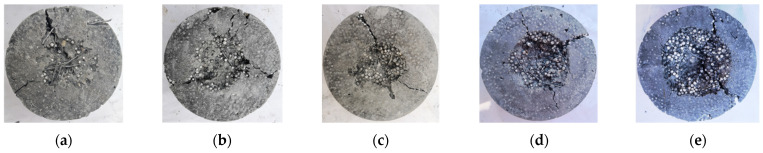
Destruction mode of SFEPSC specimens: (**a**) S1E10; (**b**) S1E20; (**c**) S1E30; (**d**) S1E40; (**e**) S1E50.

**Figure 17:**
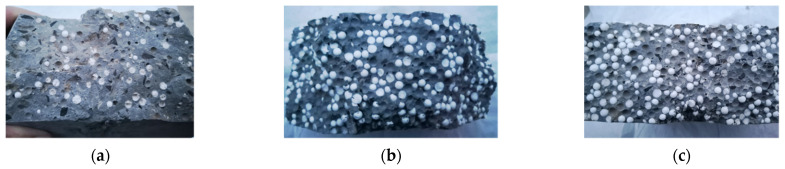
Failure fracture surface mode of EPSC specimens: (**a**) S0E20; (**b**) S0E30; (**c**) S0E40.

**Figure 18:**
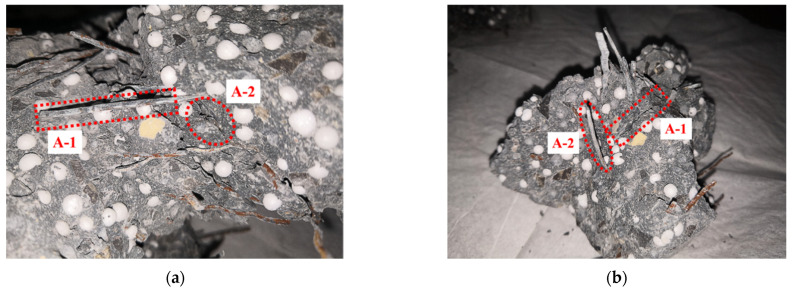
S1E20 failure mode: (**a**) A-1 and A-2 of the major specimen are the SF and SF hole, respectively; (**b**) the A-1 and A-2 of crushed specimen block are the SF hole and SF, respectively.

There are two main types of damage on the surface of specimens after impact: splitting and pitting. Figure 15 and Figure 16 show the destruction mode of EPSC and SPESC, respectively. The EPSC specimens are broken with shallow pits shown in Figure 15. The depression on the surface of the specimen is unobvious, and the failure surface is relatively flat when *V*_eps_ < 30%, as shown in Figure 17a. The pit on the specimen surface deepens when *V*_eps_ ≥ 30% and its failure surface becomes relatively rough, as shown in Figure 17b,c. The SFEPSC specimens are broken with deep pits, as shown in Figure 16. The fragments of the specimen are connected by SFs, and the failure surfaces are relatively rough, as shown in Figure 18. The pit on the surface of the specimen is relatively shallow when the *V*_eps_ < 30%, and there are randomly distributed SF connections on the pit surface. The specimen surface was locally squeezed, large deformation occurred, and the SF bounced away. The specimen was dented and destroyed along the direction of force contact surface gradually transferred to the transmission direction, and the pit depth increased with increasing *V*_eps_.

It can be seen that the specimen stiffness was larger and the pit was shallower at a smaller *V*_eps_. The overall specimen stiffness was small, and the pit was deeper at a large *V*_eps_. The SF effectively connected EPSC fragments to improve their impact resistance, which was consistent with the role of SF in normal concrete.

### 4.2. Energy Consumption Mechanism

Splits and pits in the specimen were the main energy dissipation methods for SFEPSC and EPSC after being subjected to an impact load. Due to the micro-elasticity of EPS particles, a “micro-spring damping” was formed in the specimen interior. Once the top of the specimen was subjected to an impact load, the EPS and the concrete hole absorbed part of the impact load. The other part of the load was transferred to the specimen bottom by EPS and concrete. The aggregate and bonded materials played a major role in the energy transfer process. The EPS particles absorbed energy and released it evenly to the surroundings with tiny potential energy and dissipated energy. Since the impact force of each drop weight occurred within 1ms and the stress was difficult to redistribute through the SFs in a short time, it caused a partial fracture on the impact surface of the specimen. At the same time, the specimen surface and pit absorbed energy through large deformation. If the *V*_eps_ was larger, the local absorbed load was higher than the energy transferred from the pit to its surroundings. The larger the volume of EPS in the range of 10~50%, the better cushioning effect it had under impact. The impact force on the specimen bottom was small, and finally, the specimen’s partial damage led to overall damage.

There was friction between the SF and concrete in the specimen. The SFEPSC mainly absorbed energy in two ways. One was that the friction between SF and concrete in the specimen overcame the impact load and converted it into heat. The other was that the EPS absorbed the load, converted it into micro-elastic potential energy, and released it uniformly. Although the overall bearing capacity of SFEPSC decreased after the first crack, there was friction between randomly distributed SFs and concrete inside the specimen. It could still continue to withstand impact load, as shown in Figure 18. This indicates that EPS and SF share the energy dissipation of SFEPSC, and the SF gives the specimen the ability to still dissipate energy after the first crack.

## 5. Conclusions

The current paper studied the fatigue impact resistance of SFEPSC and EPSC by a drop-weight test and statistical analysis when the *V*_eps_ was between 10% and 50%, and the following conclusions could be drawn:

1. The apparent density of the two types of concrete specimens had a linear relationship with *V*_eps_ and compressive strength. The compressive strength had a quadratic relationship with *V*_eps_. The apparent density and compressive strength of SFEPSC were higher than EPSC at the same volume of EPS;
2. By adding SF to EPSC, the impact resistance of SFEPSC was higher than EPSC. It had a highly linear relationship between the first visible crack, *N*_1_, and the ultimate failure, *N*_2_, and S1E20 had the best impact resistance;
3. The log-normal distribution and the two-parameter Weibull distribution could better describe the impact resistance of the first visible crack and the ultimate failure of SFEPSC and the EPSC at the first visible crack;
4. Under different failure probabilities, the impact resistance of SFEPSC had a concave quadratic relationship with *V*_eps_, while EPSC had a convex quadratic relationship. The impact resistance of both types could be tested and predicted by the *P* − *V*_eps_ − *lgN* curve;
5. The failure modes of the two types of concrete specimens were different. By adding SF, the pits of EPSC specimens became deepened before splitting. The pit depth of both specimens increased with the increase in *V*_eps_, and the fractures were relatively rough;
6. The energy consumption mechanism of both types of concrete specimens was different. EPSC dissipated shock loads by the EPS particles. By adding SF to EPSC, especially after the first cracking of the specimen, the SF energy absorption and friction energy dissipation characteristics were more obvious.

## Figures and Tables

**Table 1:** Chemical composition of cement and microsilica (by mass).

Oxide	SiO_2_	Al_2_O_3_	Fe_2_O_3_	CaO	MgO	Na_2_O	K_2_O	SO_3_	NaO	Loss
PC (%)	21.60	4.13	4.72	64.44	2.06	0.11	0.56	0.74	-	1.64
Ms (%)	94.43	0.93	0.97	0.28	0.77	-	-	-	1.39	1.23

**Table 2:** Physical and mechanical property of coarse aggregates (kg/m^3^).

Particle Size/mm	Apparent Density	Bulk Density	Mud Content	Crush Index/%
<10	2490	1370	0.57	7.9

**Table 3:** Mix proportion of SFEPSC and EPSC.

Type	W/B	W (kg)	Binders (kg)		FA (kg)	CA (kg)	PS (kg)	SF (%)	EPS (kg)	Slump (mm)	*ρ*_*d*_ (kg/m^3^)
			PC	Ms							
S0E0	0.44	238.2	487.2	54.1	230	536	2.8	-	-	115	1568
S1E10	0.44	238.2	487.2	54.1	230	536	2.8	78.5	2.8	71	1495
S1E20	0.44	238.2	487.2	54.1	230	536	2.8	78.5	3.1	84	1320
S1E30	0.44	238.2	487.2	54.1	230	536	2.8	78.5	10.7	98	1174
S1E40	0.44	238.2	487.2	54.1	230	536	2.8	78.5	16.7	117	1016
S1E50	0.44	238.2	487.2	54.1	230	536	2.8	78.5	25	124	857
S0E10	0.44	238.2	487.2	54.1	230	536	2.8	-	2.8	110	1413
S0E20	0.44	238.2	487.2	54.1	230	536	2.8	-	3.1	116	1257
S0E30	0.44	238.2	487.2	54.1	230	536	2.8	-	10.7	123	1115
S0E40	0.44	238.2	487.2	54.1	230	536	2.8	-	16.7	129	958
S0E50	0.44	238.2	487.2	54.1	230	536	2.8	-	25	135	805

**Table 4:** Impact resistance test results for SFEPSC and EPSC specimens (blows).

Number	*N*_1_/*N*_2_					Δ*N*				
	S1E10	S1E20	S1E30	S1E40	S1E50	S1E10	S1E20	S1E30	S1E40	S1E50
1	36/52	24/42	57/66	62/69	61/75	16	18	9	7	14
2	20/22	38/57	21/28	75/83	34/43	2	19	7	8	9
3	49/78	13/28	39/48	10/19	33/41	29	15	9	9	8
4	28/59	21/52	60/68	54/59	80/91	31	31	8	5	11
5	101/109	59/73	23/34	41/48	37/42	8	14	11	7	5
6	34/40	97/104	41/50	89/98	51/60	6	7	9	9	9
7	69/79	57/83	33/45	66/69	62/73	10	26	12	3	11
8	60/84	44/61	25/33	50/61	47/55	24	17	8	11	8
9	39/54	47/59	69/77	58/64	31/43	15	12	8	6	12
10	69/84	63/79	27/33	43/55	36/44	15	16	6	12	8
11	82/97	57/66	87/96	17/24	34/42	15	9	20	7	8
12	57/69	51/59	32/41	43/52	34/41	12	8	9	9	7
**Number**	**S0E10**	**S0E20**	**S0E30**	**S0E40**	**S0E50**	**S0E10**	**S0E20**	**S0E30**	**S0E40**	**S0E50**
1	21/22	16/17	3	3	9/12	1	1	0	0	3
2	2	3	6	5	12/15	0	0	0	0	3
3	3	4	6	5	7	0	0	0	0	0
4	2	6	7/8	6	7	0	0	1	0	0
5	4	7/8	7	15/17	9	0	1	0	2	0
6	6	7/8	17/18	7	13/15	0	1	1	0	2
7	6	7/9	9	8	11/12	0	2	0	0	1
8	11/12	10/11	11/12	8	8/9	1	1	1	0	1
9	3	9	13/15	10/12	8	0	0	2	2	0
10	4	6/7	7	7	7	0	1	0	0	0
11	2	4	2	5	4	0	0	0	0	0
12	5	6	24/25	9/10	6	0	0	1	1	0

For the sake of comparison, the number of EPSC specimens completely destroyed at the first visible crack was defined as *N*_1_.

**Table 5:** Linear regression parameter values of SFEPSC and EPSC.

Specimen Type	Rank	*a*	*b*	*R* ^2^
S1E10	12	0.9619	17.297	0.8717
S1E20	12	0.8368	23.767	0.9056
S1E30	12	0.9993	8.7812	0.9941
S1E40	12	0.9796	8.7839	0.9876
S1E50	12	1.085	5.3405	0.9854
S0E10	2	/	/	/
S0E20	6	0.5589	4.3181	0.4356 *
S0E30	5	0.9916	1.3206	0.9952
S0E40	3	/	/	/
S0E50	5	1.1163	0.7674	0.8505

Rank represents the number of valid test results. *R*^2^ = coefficient of determination. * Low precision, not included in analysis.

**Table 6:** Statistical analysis results of impact test of SFEPSC and EPSC (blows).

Statistical Parameters	*N*_1_/*N*_2_					Δ*N*				
	S1E10	S1E20	S1E30	S1E40	S1E5`0	S1E10	S1E20	S1E30	S1E40	S1E50
Rank	12/12	12/12	12/12	12/12	12/12	12	12	12	12	12
$\bar{x}$	54/69	48/64	43/53	51/58	45/54	15	16	10	7	9
*σ*	24/25	22/20	21/21	22/22	16/17	9	7	4	3	2
*COV*%	44/36	45/31	49/39	43/38	35/31	60	44	40	43	22
	**S0E10**	**S0E20**	**S0E30**	**S0E40**	**S0E50**	**S0E10**	**S0E20**	**S0E30**	**S0E40**	**S0E50**
Rank	12/2	12/6	12/5	12/3	12/5	2	6	5	3	5
$\bar{x}$	6/17	8/10	9/20	7/13	8/13	3	3	2	4	3
*σ*	5/7	4/4	6/6	3/4	3/3	0.4 ^┌^	0.7 ^┌^	0.7 ^┌^	0.8 ^┌^	1.2 ^┌^
*COV*%	83/41	50/40	66/30	43/31	38/23	13	23	35	20	40

^┌^ Qualitative analysis by score.

**Table 7:** Values of log-normal parameters for fatigue life of SFEPSC.

Blows	Specimen Type	Rank	*α*_1_	*β*_1_	*R* ^2^
*N*_1_	S1E10	12	1.7699	6.8763	0.981
	S1E20	12	1.4526	5.4318	0.9044
	S1E30	12	1.8015	6.5863	0.9672
	S1E40	12	1.2228	4.6319	0.8114
	S1E50	12	2.536	9.5326	0.8723
*N*_2_	S1E10	12	1.8087	7.5191	0.8802
	S1E20	12	2.4043	9.8664	0.9202
	S1E30	12	2.1558	8.3488	0.9657
	S1E40	12	1.6411	6.5333	0.8409
	S1E50	12	2.711	10.715	0.8253
Δ*N*	S1E10	12	1.0574	2.6636	0.8838
	S1E20	12	1.8639	4.9994	0.9736
	S1E30	12	2.5355	5.6296	0.8299
	S1E40	12	2.1484	4.2773	0.8922
	S1E50	12	2.9983	6.5431	0.9297

**Table 8:** Values of log-normal parameters for fatigue life of EPSC.

Blows	Specimen Type	Rank	*α*_1_	*β*_1_	*R* ^2^
*N*_1_	S0E10	12	1.1292	1.6642	0.9114
	S0E20	12	1.631	3.1423	0.9615
	S0E30	12	1.2035	2.4456	0.9503
	S0E40	12	2.0013	3.832	0.9480
	S0E50	12	2.5494	5.3148	0.9392
*N*_2_	S0E10	2	/	/	/
	S0E20	6	1.3246	3.6669	0.7554 *
	S0E30	5	1.0304	3.5533	0.9871
	S0E40	3	/	/	/
	S0E50	5	2.0239	5.8892	0.9199
Δ*N*	S0E10	2	/	/	/
	S0E20	6	1.2931	0.5297	0.5594 *
	S0E30	5	1.1369	0.638	0.6292 *
	S0E40	3	/	/	/
	S0E50	5	0.734	0.3714	0.8348 *

* Low precision, not included in analysis.

**Table 9:** Values of Weibull parameters for fatigue life of SFEPSC.

Blows	Specimen Type	Rank	*α*_2_	*β*_2_	*R* ^2^
*N*_1_	S1E10	12	2.1463	8.8421	0.9895
	S1E20	12	1.8045	7.2513	0.9538
	S1E30	12	2.1105	8.2194	0.9071
	S1E40	12	1.5533	6.3873	0.8947
	S1E50	12	2.8833	11.341	0.7705
*N*_2_	S1E10	12	2.2727	9.9512	0.9497
	S1E20	12	2.9517	12.638	0.9512
	S1E30	12	2.5281	10.294	0.9075
	S1E40	12	2.0628	8.7153	0.9078
	S1E50	12	3.0452	12.54	0.7116
Δ*N*	S1E10	12	1.3215	3.8322	0.9432
	S1E20	12	2.2399	6.5114	0.9609
	S1E30	12	2.9144	6.9744	0.7493
	S1E40	12	2.677	5.8332	0.9467
	S1E50	12	3.6184	8.3999	0.9254

**Table 10:** Values of Weibull parameters for fatigue life of EPSC.

Blows	Specimen Type	Rank	*α*_2_	*β*_2_	*R* ^2^
*N*_1_	S0E10	12	1.297	2.4149	0.8217
	S0E20	12	1.9356	4.2325	0.9254
	S0E30	12	1.4566	3.4633	0.9513
	S0E40	12	2.4008	5.1005	0.9323
	S0E50	12	3.1015	6.9694	0.9499
*N*_2_	S0E10	2	/	/	/
	S0E20	6	1.9619	5.7343	0.6989 *
	S0E30	5	1.6363	5.8544	0.9769
	S0E40	3	/	/	/
	S0E50	5	3.2481	9.6494	0.9298
Δ*N*	S0E10	2	/	/	/
	S0E20	6	2.1052	1.066	0.6252 *
	S0E30	5	1.8973	1.2126	0.6851 *
	S0E40	3	/	/	/
	S0E50	5	1.1474	0.8118	0.8007 *

* Low precision, not included in analysis.

**Table 11:** Fatigue lives of SFEPSC and EPSC corresponding to different failure probabilities.

Blows	Failure Probability	Log-Normal Distribution					Weibull Distribution				
		S1E10	S1E20	S1E30	S1E40	S1E50	S1E10	S1E20	S1E30	S1E40	S1E50
***N*_1_**	0.05	19	14	16	12	22	9	5	7	4	13
	0.10	24	17	19	15	26	14	9	11	7	18
	0.15	27	20	22	19	29	17	12	14	10	21
	0.20	30	24	24	22	31	21	15	17	13	24
	0.25	33	26	27	25	33	24	18	19	16	26
	0.30	36	29	29	29	35	27	21	22	19	29
***N*_2_**	0.05	26	31	22	20	28	12	17	12	9	17
	0.10	31	36	27	25	32	18	24	17	14	23
	0.15	36	39	30	28	36	23	28	21	18	27
	0.20	40	43	33	32	38	28	32	24	21	30
	0.25	44	46	35	36	41	32	36	27	25	33
	0.30	48	49	38	39	43	36	39	30	29	36
		**S0E10**	**S0E20**	**S0E30**	**S0E40**	**S0E50**	**S0E10**	**S0E20**	**S0E30**	**S0E40**	**S0E50**
***N*_1_**	0.05	1.8	3.8	3.5	4.2	5.6	0.7	1.9	1.4	2.4	3.6
	0.10	2.4	4.6	4.5	4.9	6.3	1.1	2.8	2.3	3.3	4.6
	0.15	2.9	5.2	5.3	5.4	6.8	1.6	3.5	3.1	3.9	5.3
	0.20	3.4	5.8	6.0	5.9	7.2	2.0	4.1	3.8	4.5	5.8
	0.25	3.8	6.3	6.8	6.3	7.6	2.5	4.7	4.6	5.0	6.3
	0.30	4.3	6.8	7.5	6.7	8	2.9	5.2	5.3	5.4	6.8

Note: EPSC data are analyzed in fractional form.

**Table 12:** The coefficients of *P* − *V*_eps_ − *lgN*_1_ curves of SFEPSC and EPSC.

Concrete Type	*P*	Log-Normal Distribution					Weibull Distribution			
		*m*	*n*	*l*	*R* ^2^		*m*	*n*	*l*	*R* ^2^
SFEPSC	0.05	4.9524	2.9085	1.5316	0.7469		7.8211	4.5077	1.3480	0.6315
	0.10	4.1660	2.4702	1.5819	0.8143		6.2630	3.6392	1.4477	0.6781
	0.15	3.6355	2.1744	1.6158	0.8785		5.3249	3.1162	1.5077	0.7231
	0.20	3.2138	1.9393	1.6428	0.8943		4.6387	2.7337	1.5516	0.7706
	0.25	2.8520	1.7377	1.6659	0.9259		4.0889	2.4271	1.5868	0.8226
	0.30	2.5272	1.5566	1.6867	0.9789		3.6236	2.1678	1.6116	0.9001
EPSC	0.05	−2.0230	−2.2338	0.1027	0.8428		−1.5322	−2.5134	−0.3475	0.8159
	0.10	−2.1575	−2.1571	0.2261	0.8940		−1.7988	−2.3615	−0.1030	0.8801
	0.15	−2.2482	−2.1054	0.3093	0.9179		−1.9592	−2.2701	0.0442	0.9194
	0.20	−2.3204	−2.0643	0.3755	0.9436		−2.0766	−2.2032	0.1519	0.9453
	0.25	−2.3822	−2.0290	0.4323	0.9348		−2.1707	−2.1496	0.2382	0.9482
	0.30	−2.4378	−1.9974	0.4833	0.9208		−2.2503	−2.1042	0.3112	0.9479

## Data Availability

Part of the data underlying this article will be shared on reasonable request from the corresponding author.
